# Intralesional injection treatment of hypertrophic scars and keloids: a systematic review regarding outcomes

**DOI:** 10.1186/s41038-015-0015-7

**Published:** 2015-08-26

**Authors:** Aurelia Trisliana Perdanasari, Matteo Torresetti, Luca Grassetti, Fabio Nicoli, Yi Xin Zhang, Talal Dashti, Giovanni Di Benedetto, Davide Lazzeri

**Affiliations:** 1 Department of Plastic and Reconstructive Surgery, Shanghai Ninth People’s Hospital, Shanghai JiaoTong University, School of Medicine, 639 Zhi Zao Ju Road, 200011 Shanghai, P.R. China; 2 Department of Plastic and Reconstructive Surgery, Marche Polytechnic University Medical School, University Hospital of Ancona, Ancona, Italy; 3 Department of Plastic Reconstructive and Aesthetic Surgery, University of Rome, Rome, Italy; 4 Plastic Reconstructive and Aesthetic Surgery Unit, Villa Salaria Clinic, Rome, Italy

**Keywords:** Intralesional injections, Hypertrophic scar, Keloid, Systematic review, Outcomes

## Abstract

**Background:**

The aim of this review was to explore the existing body of literature focusing on the intralesional treatments of keloids and hypertrophic scars.

**Methods:**

A comprehensive systematic review of related articles was conducted across multiple databases. Article selection was limited to those published in the English language between 1950 and 2014. Search terms for the on-line research were “scar(s),” “keloid(s),” “hypertrophic,” “injection,” “intralesional,” and “treatment”.

**Results:**

The initial search returned 2548 published articles. After full text review, the final search yielded 11 articles that met inclusion criteria. A total of 14 patient samples in 11 articles were collected. The most frequent intralesional injection treatment studied was triamcinolone (*n* = 5), followed by bleomycin (*n* = 3), 5-fluorouracil (*n* = 2), verapamil (*n* = 2), cryosurgery, and collagenase. The scar height reduction for all but one study was demonstrated, with acceptable complication and recurrence rate. Only three articles reported a follow-up period longer than 18 months, and only two studies used standardized outcome criteria with a quantitative scale.

**Conclusions:**

Although many treatment options have already been described in the literature, there is no universally accepted treatment resulting in permanent hypertrophic or keloid scar ablation. The lack of adequately long-term powered randomized controlled trials does not permit to establish definitive conclusions with implications for routine clinical practice.

**Level of evidence:**

III/Therapeutic

## Background

The cicatrization results in a spectrum of scar formation ranging from nearly scarless healing to excessive fibrosis or atrophy. Pathological scars can be basically divided into two main categories: (1) keloidal and hypertrophic scars and (2) atrophic scars. While hypertrophic scars are confined to the original injury and increase in size by pushing outward and not by invasion, keloids are characterized by scar tissue that extends beyond the confines of the original wound. Hypertrophic scars usually grow quickly (3–6 months) and after this period can partially regress. On the contrary keloids have a long (years), permanent, and uninterrupted evolution. The incidence of hypertrophic scars and keloids varies with age, race, sex, anatomic location, and the inciting trauma. Associated symptoms such as pruritus, dysesthesia, and pain, as well as restricted range of motion and contracture formation may be observed with both keloids and hypertrophic scars but tend to be more prevalent with keloids [1, 2].

Historically, a variety of treatment approaches for keloids and hypertrophic scars have been extensively described in the literature. The methods are ranging from surgical to non-surgical methods. Evidence supports occlusive dressings, compression therapy, silicone sheeting, intralesional corticosteroid injections, cryotherapy, surgical removal, pulsed dye laser, radiation, imiquimod cream, intralesional verapamil, 5-fluorouracil, bleomycin, and interferon alfa-2b injections [3, 4]. In some cases, when surgical approaches are inadvisable, intralesional injection treatments play an important role in the treatment of keloids.

Despite the large number of described techniques, scar therapy is still challenging and controversial with a high recurrence rate regardless of therapy (especially for keloids). Through the literature retrieval, we have found most of the literature that is available about the intralesional injection treatment of hypertrophic scars and keloids. The aim of this article is to systematically review the existing body of literature regarding the management of hypertrophic scars and keloids with intralesional injections.

## Methods

### Search strategy

A comprehensive systematic review of related articles was conducted in January of 2014 using databases including Medline, the Cochrane database, Google and Google Scholar, Clinical Trials.gov, Current Contents, and PubMed. Article selection was limited to those published in the English language between January 1, 1950, and January 15, 2014. Search terms for the on-line research were a combination of the following: “scar(s),” “keloid(s),” “hypertrophic,” “injection,” “intralesional,” “treatment”. Reference lists of selected articles, other related studies, and review articles were examined for eligible studies. A cross-referencing from identified articles and conference abstracts was also performed. Numerous articles were identified through searches of the extensive files of the authors. Abstracts and reports from meetings were included only when they related directly to previously or subsequently published work.

### Selection criteria

Three researchers (A.T.P., M.T., and D.L.) performed the review process for inclusion in the initial review, and the senior author (Y.X.Z.) acting as an arbiter solved all the disagreement between them during the procedure. General inclusion criteria consisted of articles discussing injection treatments related to scar(s) and their outcomes. The initial search returned 2548 published articles.

Specific inclusion criteria mandated retrospective or prospective investigations that met all the following criteria: 1. Papers published in the English language; 2. clinical investigation assessing single-substance injection treatment for hypertropic or keloid scars; 3. more than 5 patients included in the study; 4. clear description of the nature of the injected substance; 5. clear description of the nature (iatrogenic, post-traumatic, burn sequel), sites, and age of the scar; 6. adequate follow-up (the average patient follow-up was limited to at least 6 months; a shorter follow-up period was considered grossly inadequate because keloids can recur from months to years after treatment).

Studies that solely discussed about injection techniques were excluded as well as those in which combined treatments (other-than-injection procedure associated to injection procedure(s)) and those investigations in which more than one substance was injected to treat the scar.

**Fig. 1:**
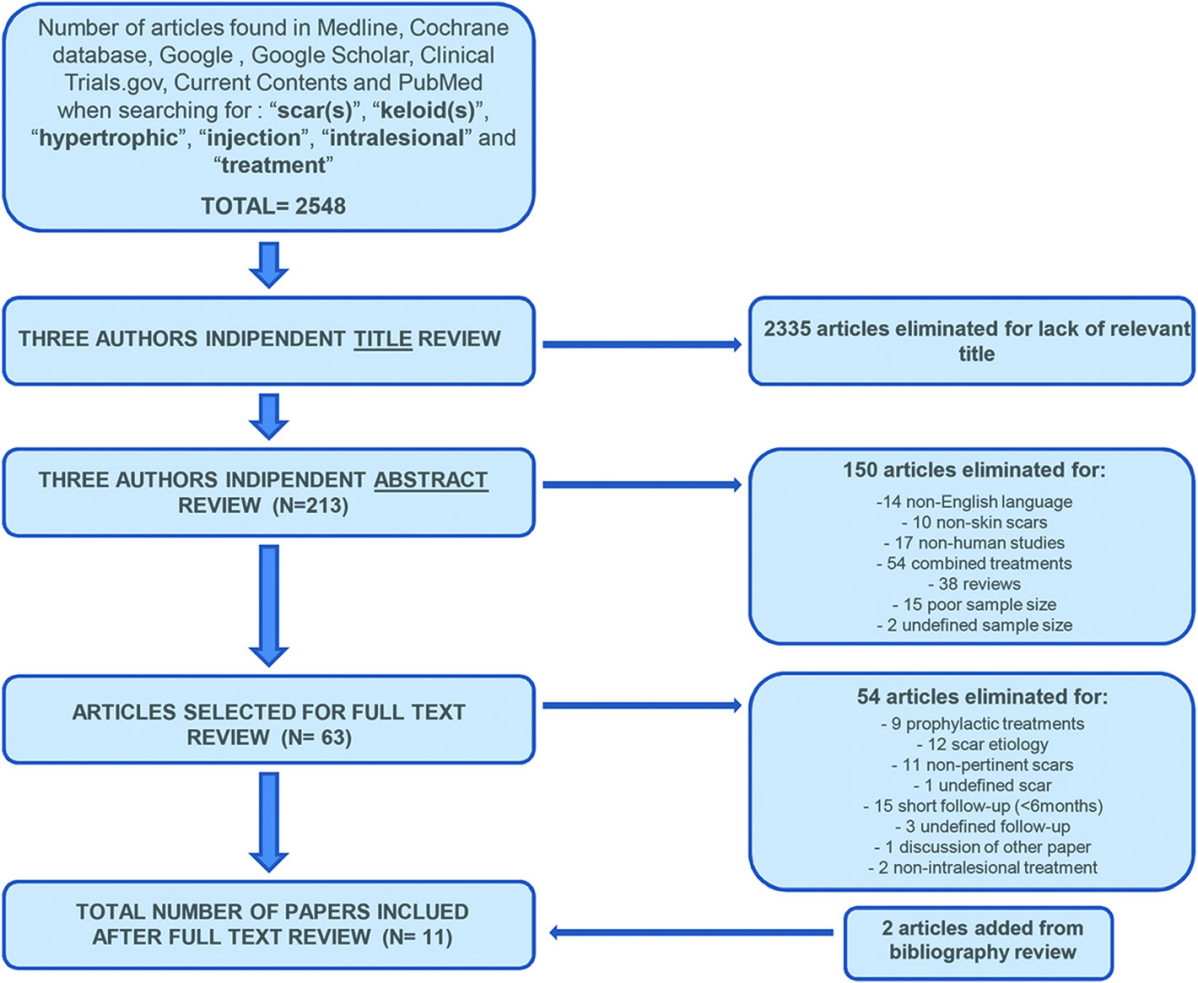
Citation attrition diagram

After exclusion, 213 articles were selected for inclusion in the initial review. A further screen excluded 150 papers because 14 of them had pertinent topic, but the manuscript was written in other than English language, 10 discussed about scar not involving the skin, 17 were related to non-human (laboratory and animal) studies, 54 presented multiple substance injections for the same scar or intralesional injection treatment combined with one or more different scar treatment (laser, surgery, RT, compressive therapy, cryosurgery and electrochemotherapy), 38 were reviews, 15 were paper describing single case report or less than 5 cases, and 2 did not report the exact number of patients included in the study. The same researchers (A.T.P., M.T., and D.L.) reviewed the remaining 63 articles in their entirety to ensure adequate data content for inclusion. This final screen excluded 54 articles because 9 discussed about prophylactic treatments for improvement of skin scarring [5–13], 12 did not adequately describe the causes of the scars [14–25], 11 presented different kinds of scars such as atrophic or depressed scars (10) [26–35] or cicatricial ectropion (1) [36] which usually requires different treatment modalities due to their different nature and characteristics, 1 did not adequately describe the type of the scar [37], 15 reported an inadequate average follow-up period (less than 6 months) [4, 38–51], 3 did not adequately report the follow-up period [52–54], 1 was a discussion of another paper (already excluded due to the poor sample population) [55], and 2 described a non-intralesional treatment (Klinger et al. [56] reported the use of autologous fat graft inserted into the dermo-hypodermic junction, while Balkin [57] described the use of fluid silicone injections under the scars). There were two articles lost in the initial search, but they were cited in the articles already included, so they were also selected for study [58, 59]. After full text review, the final search yielded 11 articles that meet inclusion criteria and are thus presented in this review for analysis and data extraction (Fig. 1 illustrates the literature review process) [58–68].

### Data extraction

Data were extracted and then reviewed, and all reported results were summarized to include common and clinically important outcomes. The following data were extracted from each primary article and used for descriptive comparisons: author, year, study design, level of evidence, mean age, sample size, number of treated scars, type of treatment (Table 1), and details regarding outcomes, including scar volume reduction, improvement of scar-related symptoms, recurrence rate, local or systemic side effects and follow-up periods (Table 2).

**Table 1:** Management of hypertrophic scars and keloids: systematic review

Reference	Sample size	Study	Randomization	Controlled	Comparative	LOE	Patients age	Number of scars	Treatment
Aggarwal H et al., 2008 [60]	50 patients	Prospective	No	No	No	IV	N.A.	50	Bleomycin
Ahuja RB and Chatterjee P, 2013 [61]	40 patients	Prospective	Yes	No	Yes	II	Average age: N.A. (range: 15–60)	22	Triamcinolone
								26	Verapamil
Espana A et al., 2001 [58]	13 patients	Prospective	No	No	No	IV	Average age: 24 years (range: 14–36)	13	Bleomycin
Griffith BH, 1966 [59]	29 patients	Retrospective	No	No	No	IV	Average age: 22 years (range: 1–58)	37	Triamcinolone
Gupta S and Kumar B, 2001 [62]	12 patients	Prospective	No	No	No	IV	Average age: 31 years (range: 19–50)	12	Cryosurgery
Kang N et al., 2006 [63]	5 patients	Prospective	No	No	No	IV	Average age: 31 years (range: 12–46)	N.A.	Collagenase
Kiil J, 1977 [64]	44 patients	Prospective	No	No	No	IV	Average age: 16 years (range: 6–46)	N.A.	Triamcinolone
Kontochristopoulos et al., 2005 [65]	20 patients	Prospective	No	No	No	IV	Average age: 30 years (range: 12–65)	N.A.	5-Fluorouracil
Manuskiatti W and Fitzpatrick RE, 2002 [66]	10 patients	Prospective	Yes	No	Yes	II	Average age: N.A. (range: 25–74)	10	5-Fluorouracil
									Triamcinolone
Margareth Shanthi FX et al., 2008 [67]	54 patients	Prospective	Yes	No	Yes	II	Average age: 20 years (range: 10–50)	N.A.	Triamcinolone
							Average age: 26 years (range: 10–50)	N.A.	Verapamil
Saray Y and Güleç AT, 2005 [68]	14 patients	Prospective	No	No	No	IV	Average age: 32 years (range: 16–73)	15	Bleomycin

**Table 2:** Outcomes and complications

Reference	Volume reduction (percent of lesions)	Scar etiology	Method of scar height assessment	Symptoms (percent of patients)	Recurrence rate	Side effects	Follow-up (months)
Aggarwal H et al., 2008 [60]	Complete flattening (44 %)	Trauma (30)	N.A.	Symptomatic relief (88.9 %)	14 % (7 patients)	Ulceration (16 %)	18
		Burn, vaccination, postoperative (10)				Pain (30 %)	
(Bleomycin)						Hyperpigmentation (14 %)	
		No particular histories (10)					
Ahuja RB and Chatterjee P, 2013 [61]	Complete flattening [VSS mean value = 0] (100 %)	Foruncle/post-infective (14)	VSS/Caliper	–	0 %	Pain (3 %)	6
		Surgery (1)				Teleangectasia (9 %)	
		Burns (4)				Skin atrophy (18 %)	
(Triamcinolone)		Trauma (3)					
(Verapamil)							
	Complete flattening [VSS mean value = 0] (100 %)	Foruncle/post-infective (16)	VSS/Caliper	–	0 %	Pain (3.5 %)	6
		Surgery (2)					
		Burns (4)					
		Trauma (4)					
Espana A et al., 2001 [58]	Complete flattening (54 %)	Surgery (5)	N.A.	Itching eliminated (100 %)	15 % (2 patients)	Hyperpigmentation (15 %)	13
(Bleomycin)		Spontaneous (3)					
		Acne (2)					
		Vaccination (2)					
		Cut (1)					
Griffith BH, 1966 [59]	Complete flattening (51 %)	Infection, surgery, vaccinations, burns, piercing, lacerations, spontaneous, varicella	N.A.	Symptoms eliminated (59 %) or reduced (41 %)	0 %	Skin atrophy (15 %)	10
(Triamcinolone)							
Gupta S and Kumar B, 2001 [62]	>75 % flattening (58 %)	Burn, acne	N.A.	Symptoms eliminated (100 %)	0 %	Hypo/depigmentation (100 %)	9
(Cryosurgery)							
Kang N et al., 2006 [63]	No changes in scar volume	Infection (1), assault (1), acne (2), coronary bypass graft (1)	Stereophotogrammetry	–	100 %	Pain (100 %)	6
						Blistering and skin ulceration (80 %)	
						Swelling and bruising (100 %)	
						Pyrexia (20 %)	
(Collagenase)							
Kiil J, 1977 [64]	Complete flattening (93 %)	Vaccination, surgery, acne, varicella, piercing, spontaneous	N.A.	Itching eliminated (93 %)	50 %	Acne, menstrual irregularity, fluid retention and striae (percent not reported)	60
(Triamcinolone)							
Kontochristopoulos et al., 2005 [65]	Complete flattening (5 %)	Acne, surgery, folliculitis, vaccination	N.A.	Itching resolution (% not defined)	47 %	Pain (100 %)	12
	75 % flattening (40 %)					Skin ulcerations (30 %)	
	50 % flattening (40 %)						
(5-Fluorouracil)	25 % flattening (10 %)					Hyperpigmentation (100 %)	
	0 % flattening (5 %)						
Manuskiatti W and Fitzpatrick RE, 2002 [66]	Significant flattening (percent of flattening not reported)	Surgery (sternotomy)	Caliper	–	–	Pain (100 %)	8
						Purpura (20 %)	
(5-Fluorouracil)							
(Triamcinolone)	Significant flattening (percent of flattening not reported)		Caliper	–	–	Pain (100 %)	8
						Hypopigmentation (20 %)	
						Telangiectasia (20 %)	
						Skin atrophy (10 %)	
Margareth Shanthi FX et al., 2008 [67]	Complete flattening [VSS scale]	Acid burns, trauma, surgery, acne, insect bite	VSS/Caliper	–	0 %	Hypo/hyperpigmentation	12
						Menstrual irregularity	
(Triamcinolone)							
(Verapamil)							
	97 % flattening [VSS scale]		VSS/Caliper	–	0 %	Profuse sweating	12
						Pain	
Saray Y and Güleç AT, 2005 [68]	Complete flattening (73.3 %)	Surgery (10), sebaceous cyst (2), vaccination, acne, trauma	Caliper	Complete resolution of pain (67 %) and itching (80 %)	0 %	Pain (50 %)	19
	>90 % flattening (6.7 %)					Ulceration (100 %)	
(Bleomycin)							
	75–90 % flattening (13.3 %)					Hyperpigmentation (29 %)	
	50–75 % flattening (6.7 %)					Skin atrophy (21 %)	

*N.A.* not available, *VSS* Vancouver Scar Scale

## Results

### Description of included studies

The articles included were published between 1966 and 2013. Sample sizes varied from 5 patients [63] to 50 patients [60]. The exact number of treated scars was not reported in four studies [63–65, 67]. One article did not provide any information on patient age [60]. In two studies, the average age was not reported [59, 64]; in the remaining nine studies, the sample population had a low average patient age, between 16 and 32 years. Patient age range was listed in all the articles, revealing ages between 1 and 74 years. Ten studies were prospective investigations [58, 60–69] and 1 was retrospective [59]. The level of evidence was II in three studies [61, 66, 67] and IV in the remaining 8 [58–60, 62–65, 68]. Eight studies described a single treatment in a single patient sample [58–60, 62–65, 68]. Two articles described two different treatments in two separate patient samples [61, 67]. One study described two different treatment methods in the same patient sample (the substances were injected in two different areas of the same scar) [66]. A total of 16 patient samples in 13 articles were collected. The most frequent treatment studied was triamcinolone (five cases) [59, 61, 64, 66, 67], followed by bleomycin (three cases) [58, 60, 68], 5-fluorouracil (5-FU; two cases) [65, 66], verapamil (two cases) [61, 67], cryosurgery [62], and collagenase [62]. All of these techniques were provided through intralesional injections.

### Outcomes, complications, and recurrences

The most commonly studied outcome measure was the scar height reduction (percentage reduction from baseline) that was usually classified by most authors by using the following scale: complete flattening (100 %), significant flattening (>75 %), moderate flattening (50–75 %), and minimal flattening (<50 %). Furthermore, the method used for scar height reduction assessment was investigated. Other studied outcome measures included the reduction of scar-related symptoms, complication rate associated with every treatment, recurrence rate, and the follow-up period. Table 2 summarizes all these data.

The scar height reduction for all but one study was demonstrated. Only Kang et al. [63] obtained no changes in scar volume after treatment of keloids and hypertrophic scars with intralesional injections of collagenase.

The use of Triamcinole was described in five cases [59, 61, 64, 66, 67] with complete scar flattening observed in four cases [59, 61, 64, 67]. The rates, where provided, ranged from 51 to 100 % of patients with mean follow-up between 6 and 60 months. One case observed a significant flattening after 8 months of follow-up without reporting the rate of flattening [66]. Of the five studies included in the review, only two provided specific data regarding the improvement of scar-related symptoms [59, 64] with rates ranging from 41 to 93 %. Most frequent side effects included pain (3 to 100 %, where provided) reported in three studies, skin atrophy (10 to 18 %) reported in three studies, hypo/hyperpigmentation (20 %, where provided) reported in two cases and telangectasia (9 to 20 %) reported in two cases. No recurrences were observed in three cases with a mean follow-up ranging from 6 to 12 months. A recurrence rate of 50 % was observed in one case with mean follow-up of 60 months. One study did not provide this data.

All the three studies describing the use of bleomycin [58, 60, 68] reported complete scar flattening with rates ranging from 44 to 73.3 %. All these studies provided data regarding the improvement of scar-related symptoms with rates ranging from 67 to 100 %. Most frequent side effects included hyperpigmentation (14 to 29 %) reported in all studies, ulceration (16 to 100 %) and pain (30 to 50 %) reported in two studies, and skin atrophy reported in one case (21 %). Low recurrence rates (14 and 15 %) were reported in two studies with mean follow-up of 18 and 13 months, respectively, while no recurrences were observed in another study after 19 months of follow-up.

The use of 5-FU was studied in two cases [65, 66]. One study reported a moderate (50 to 75 %) scar flattening in 80 % of patients; another study reported significant flattening without providing the exact rate of flattening. One investigation obtained complete resolution of scar-related symptoms, whereas the second study did not provide this data. Most frequent side effects included pain (100 %) reported in both the studies and skin ulcerations (30 %) reported in one investigation. The recurrence rate was reported only in one paper (47 %) with a mean follow-up of 12 months.

Verapamil was used in two cases [61, 67]; one study reported a complete scar flattening in all the patients, while the other study obtained a near total (97 %) scar flattening (rate of patients not available). Both studies did not provide any data regarding scar-related symptom improvement. The most frequent side effect was pain reported in both studies (3.5 %, where provided). No recurrences were observed after 6 and 12 months of follow-up.

Cryosurgery was reported in one study [62] with significant (>75 %) scar flattening in 58 % of patients. This study reported complete symptom resolution in all patients, with constant side effect (hypo-depigmentation) in all treated patients. No recurrences were observed after 9 months of follow-up.

One article studied the use of collagenase [63], revealing this treatment as ineffective. Indeed, it reported no changes in scar volume after 6 months follow-up. Data regarding symptom resolution were not provided, while several side effects (pain, blistering, skin ulceration, swelling, and bruising) were observed in most patients.

## Discussion

Keloids and hypertrophic scars represent an exuberant healing response that poses a challenge for physicians; in severe cases, they can produce dramatic cosmetic deformity and occasional functional problems. Although a multitude of options exist in the literature on scar intralesional therapy, currently no single therapy has consensus approval. The literature itself is confusing because there is no standardized method of reporting results and as a clinician, sometimes it is really difficult to compare results of several treatment options in a truly scientific manner [17].

Some important aspects emerged from this systematic review. In several articles, the number of patients investigated is slightly small: only in two manuscripts [60, 64] the number of patients was more than 30 and only in four articles is more than 20 [59, 60, 64, 67]. This lack of large cohort studies is unexplainable considering that intralesional treatment of hypertrophic scars and keloids is quite common in the clinical practice and it is usually performed in the outpatient office without requiring surgery and hospitalization and thus is relatively inexpensive. Furthermore, five articles did not provide any data regarding the improvement of scar-related symptoms, and four patient samples in three studies did not report the exact rate of scar flattening, which are considered two of the main outcome measures of scar treatment. Only five articles reported the exact method used for scar height measuring [61, 63, 66–68] and only two used the Vancouver Scar Scale which is the standard scale used universally for scar assessment [69].

Another important consideration was that nine patient samples in seven articles had no scar recurrence and two other samples had low recurrence rate (15 %). Nevertheless, in all of these studies, the follow-up period ranged from 6 to 18 months that is slightly inadequate because keloids can recur from months to years after treatment. Therefore, a 6-month-long period of observation cannot predict the scar’s tendency for hypertrophy in a long term. It is therefore possible that the low recurrence rate observed in these studies could simply be related to the short follow-up period. Indeed, the absence of recurrences observed by Ahuja and Chatterjee [61] and by Griffith [59] with triamcinolone after 6 and 10 months of follow-up, respectively, grossly disagrees with data collected by Kiil [64] that reported a high recurrence rate (50 %) after 60 months of follow-up. Furthermore, Nanda and Reddy [68] reported no recurrences with 5-FU after 6 months of follow-up, while Kontochristopoulos et al [65] observed a high recurrence rate (47 %) after 12 months of follow-up.

### Limitation of the current investigation

The systematic review reported here combined data across studies in order to estimate the effects of intralesional treatments of hypertrophic scars and keloids with more precision than is possible in a single study. The main limitation of this systematic review is that the quality of the included papers varied. The level of evidence of the included papers was II only in 3 studies [61, 66, 67] and IV in the remaining included investigations. Indeed, 3 papers described randomized uncontrolled comparative trials [61, 66, 67], whereas the other 8 reported one-treatment series without comparative or controlled groups. None of the articles explicitly stated that analysis of data adhered to the intention-to-treat principle, which could lead to an overestimation of treatment effect in these trials. The sample sizes were too small in all papers to allow for an objective evaluation of treatment effectiveness with confidence. All studies had methodological shortcomings and most lacked of appropriate statistical methods. Although systematic reviews including only randomized control trials may offer the best potential evidence, it is possible that this method may neglect data available from other papers. Therefore, the inclusion of both randomized and nonrandomized trials collectively has provided further information at the expense of the level of evidence in this systematic review.

### Implications for research and future perspective

As the treatment of keloids and hypertrophic scars is still a challenge, it is clear that an adequate clinical evaluation of these lesions before and after a treatment by using standardized models is necessary. To best serve the body of the literature on this topic, future studies regarding intralesional treatments of keloids and hypertrophic scars should have a focus on the following principles. First, larger sample size, randomized, and placebo-controlled studies are warranted. Second, the inclusion criteria should become stricter because of the needing of highly selected patients according to medical history, previous treatments, and site of the scar, with a clear distinction between the nature (iatrogenic, traumatic, or burn causes) and the type of the scar (keloid and hypertrophic scars). Third, more explicit details about the assessment of the clinical outcomes should be always reported, both for the volume reduction and for the scar-related symptoms improvement. Fourth, the results should be based on a set of standardized outcome criteria with quantitative scales and methods of outcome evaluation to make the results comparable. Fifth, the follow-up period should always be provided and, if possible, should always be longer as possible (at least 24 months) due to the high recurrence rate of these types of lesions, especially for keloids.

Although the investigation and application of preventive measures remain a priority before, during, and immediately after wound closure, future researches are required to determine the mechanism of action for different injectable substances and to examine the efficacy of as many quantifiable and objectivable parameters as possible. In addition, further potential treatment approaches in the field of intralesional non-injective therapy alone such as nanotechnologies and molecular therapies should be investigated in the clinical daily practice.

## Conclusions

The present systematic review summarized the current evidence on the effectiveness of intralesional treatment for keloid and hypertrophic scar. Although many treatment options have already been described in the literature, there is no universally accepted treatment resulting in permanent hypertrophic or keloid scar ablation. No definitive conclusions with implications for routine clinical practice could be highlighted regarding the effectiveness of different techniques, due to the scarcity and low quality of the included studies. Despite several studies demonstrating encouraging results, the lack of the abovementioned criteria makes it difficult to establish the standard practice and identify the best technique ensuring long-term effectiveness and low recurrence rates. Therefore, adequately powered randomized controlled trials should be recommended with an improvement of the quality on the effectiveness of the intralesional injection therapies for hypertrophic and keloid scars to better clarify these aspects.
